# 硬化性肺泡细胞瘤35例临床特征分析

**DOI:** 10.3779/j.issn.1009-3419.2020.103.19

**Published:** 2020-12-20

**Authors:** 小静 刘, 志豪 黄, 建勇 张

**Affiliations:** 563000 遵义，遵义医科大学附属医院呼吸与危重症医学科呼吸二病区 The Second Department of Pulmonary and Critical Care Medicine, Affliated Hospital of Zunyi Medical University, Zunyi 563000, China

**Keywords:** 肺硬化性血管瘤, 硬化性肺泡细胞瘤, 良性肿瘤, Pulmonary sclerosing hemangioma, Pulmonary sclerosing pneumocytoma, Benign tumor

## Abstract

**背景与目的:**

硬化性肺泡细胞瘤（pulmonary sclerosing pneumocytom, PSP）是临床上相对少见的良性肺肿瘤，多发于中年女性，无典型的临床表现及影像学表现。PSP包括表面立方上皮细胞和圆形间质细胞2种基本细胞类型，有4种组织学类型（出血性、硬化性、实性和乳头状），可发生远处转移，但生长缓慢，术前病理检查易误诊。本研究通过探讨该病的临床特征，旨在提高临床医师对PSP的认识。

**方法:**

回顾性分析我院2011年1月-2019年12月收治的35例经病理学检查确诊的PSP临床病例资料。

**结果:**

本研究共35例患者，男性12例，女性23例，平均年龄51岁。7例因体检或常规行胸部计算机断层扫描（computed tomography, CT）偶然发现，28例因咳嗽、咳痰、咯血、胸痛等症状就诊。影像学主要表现为肺部孤立的、边界清楚的圆形或类圆形结节、肿块影。本组有12例行经皮肺穿刺组织病理活检术，仅有7例诊断为PSP。本组共28例行手术治疗，24例术中行快速冷冻病理切片检查，仅5例提示PSP。术后病理检查结果1例诊断肺角化型鳞癌伴局部PSP，其余均诊断为PSP。本组手术和非手术患者出院后随访1年-8年，总体恢复好，复查胸部CT无复发和转移。

**结论:**

PSP为临床上少见的肺部良性肿瘤，多发于中年女性，临床表现、影像学检查均缺乏特异性，经皮肺穿刺病理检查及术中快速冰冻病理切片容易误诊，多数病例最终需术后病理检查才能明确诊断。

## Abstract (en)

**Background and objective:**

Pulmonary sclerosing pneumocytoma (PSP) is rare benign lung tumor which usually develops in middle-aged women without typical clinical and imaging findings. PSP consists of two basic cell types (surface cubic epithelial cells and round mesenchymal cells) and four histological types (hemorrhagic, sclerotic, solid and papillary). It grows slowly, but it can metastasize to distant organs. The pathology before surgery is easily misdiagnosed. This study aims to improve clinicians' understanding of PSP by discussing the clinical characteristics of the disease.

**Methods:**

This represents a retrospective study of thirty-five patients diagnosed with pulmonary sclerosing pneumocytoma by pathological examination from January 2011 to December 2019.

**Results:**

A total of 35 patients in this study, 12 cases were male and 23 cases were female, the average age is 51 years old. 7 cases were discovered accidentally by physical examination or routine chest computed tomography (CT), and 28 cases were found due to symptoms such as cough, sputum, hemoptysis and chest pain. The imaging changes is mainly featured with isolated or clear circular or round-like single nodule and lump in the lungs. In this group, 12 cases underwent percutaneous lung biopsy, only 7 cases were diagnosed with PSP. A total of 28 patients underwent surgery, 24 cases underwent rapid frozen pathological biopsy, only 5 cases diagnosed with PSP. Postoperative pathological examination results shows that 1 case was diagnosed with keratotic squamous cell carcinoma with partial PSP, and the rest were diagnosed with PSP. The surgical and non-surgical patients were followed up for 1 to 8 years after discharge, and the overall recovery was good. The patients were no recurrence and metastasis on chest CT review.

**Conclusion:**

PSP is a clinically rare benign lung tumor, which is more common in middle-aged women. The clinical manifestations and imaging features are lack of significance. Percutaneous lung puncture pathological examination and intraoperative rapid frozen pathological sections often leads to misdiagnosis. Final diagnosis relies on postoperative pathological work-up for most cases.

硬化性肺泡细胞瘤（pulmonary sclerosing pneumo-cytoma, PSP）是临床上相对少见的良性肺肿瘤，临床表现缺乏特异性，常见症状有咳嗽、咳痰、胸痛、咯血等，少见表现有胸闷、发热等，尚有不少患者无症状而于体检时偶然发现。该病典型的影像学表现为边界清楚的圆形或类圆形结节、肿块影，无特异性；术前胸部计算机断层扫描（computed tomography, CT）检查及术中冰冻切片病理误诊率较高，通常需行手术切除后行病理检查以明确诊断。本研究通过回顾性分析35例PSP的临床特征，以提高临床医师对该疾病的认识。

## 1 资料与方法

### 1.1 基本资料

本研究共35例PSP患者，男性12例，女性23例，男女比约为1:1.92；年龄16岁-71岁，平均51岁。表 1主要呈现了35例PSP的CT特征。

**表 1:** 硬化性肺泡细胞瘤的CT特征 CT characteristics of pulmonary sclerosing pneumocytoma

Patient No.	Age (yr)	Gender	Site	Tumor number	Tumor edge	Tumor size (mm)	Conclusion
1	62	F	LLL	1	Clear	50×37	LN
2	63	M	LLL	1	Blur	54×49	Ca
3	48	F	RLL	1	Clear	29×25	LN
4	44	F	LLL	1	Clear	20×19	LN
5	66	F	LUL	1	Clear	15×12	LN
6	54	F	RUL	2	Clear	45×36	LN
7	47	F	RML	1	Clear	15×16	LN
8	53	F	LLL	1	Clear	19×22	LN
9	61	F	LLL	1	Clear	35×40	PSP
10	48	M	LUL	1	Clear	52×36	Ca
11	70	M	LLL	1	Blur	45×30	Inflammatory lumps
12	58	F	RLL	1	Clear	38×35	LN
13	43	F	LUL	1	Clear	18×15	LN
14	16	M	LUL	1	Clear	33×30	TB
15	66	F	RUL	1	Clear	49×50	LN
16	54	M	RLL	1	Blur	45×22	Ca
17	71	F	LLL	1	Clear	NA	Inflammatory lesions
18	50	F	RML	2	Clear	30×25	PSP
19	54	F	RML	1	Clear	35×35	PSP
20	58	M	LUL	> 2	Blur	NA	LN
21	45	M	LUL	1	Clear	52×41	LN
22	60	M	LUL	1	Blur	38×36	Ca
23	39	F	LLH	1	Clear	NA	Bronchocyst
24	59	F	LLL	1	Clear	51×50	Ca
25	60	F	LLL	1	Clear	NA	LN
26	56	M	RLL	1	Blur	33×45	Inflammatory lesions
27	41	M	RUL	> 2	Clear	NA	LN
28	33	F	RUL	1	Clear	29×25	LN
29	52	F	LLL	1	Clear	40×31	LN
30	45	F	LLL	1	Clear	49×37	LN
31	50	F	RLL	1	Clear	40×31	Inflammatory lesions
32	46	F	RML	1	Clear	15×12	LN
33	43	M	RML	2	Clear	13×11	LN
34	69	M	RML	1	Clear	54×51	Ca
35	41	F	LLL	1	Clear	38×39	Ca
F: female; M: male; LLL: left lower lobe; RLL: right lower lobe; LUL: left upper lobe; RUL: right upper lobe; RML: right middle lobe; LLH: left lung hilum; NA: not available; LN: lung nodules; Ca: cancer; CT: computed tomography; TB: tuberculosis.							

### 1.2 临床表现

有咳嗽、咳痰共17例，以咳嗽、咳痰为单发表现4例，伴有胸闷1例，伴有气促2例，伴有痰中带血2例，伴有咯血4例，伴有胸痛5例；以气促为单发表现1例；以咯血为单发表现1例；以胸痛为单发表现6例；胸闷伴胸痛1例，胸闷伴气促1例；干咳伴背部疼痛有1例；入院常规行胸部CT时发现肺部占位性病变6例，体检时发现肺部团块影1例。

### 1.3 辅助检查

本组术前有16例行血清肿瘤标志物检查，包括癌胚抗原（carcinoembryonic antigen, CEA）、鳞状细胞相关抗原（squamous cell carcinoma antigen, SCC）、胃泌素释放肽前体（pro-gastrin-releasing peptide, Pro-GRP）、神经元烯醇酶（neuron-specific enolase, NSE）、细胞角蛋白19的可溶性片段（cytokeratin 19 fragment, CYRA21-1）均未见异常。本研究35例患者胸部CT均为阳性（图 1-图 4），病变发生在左肺20例（上叶7例，下叶12例，肺门1例）、右肺15例（上叶4例，中叶6例，下叶5例）；有3例患者提示有2个肺结节，2例患者提示双肺多发小结节，其余均为单发结节；29例CT表现为边界清楚的圆形或类圆形结节、肿块影，6例表现为不规则边缘模糊的密度增高影，密度欠均匀。病灶直径为11 mm-54 mm，平均直径约34 mm。伴有单侧少量胸水8例，双侧少量胸水2例，纵隔淋巴结增大4例。行胸部CT增强扫描共11例，4例强化不均匀，其余均为均匀性强化。最终CT结果仅有3例提示PSP，7例提示肺癌，4例提示炎性病变，1例提示支气管囊肿可能性大，1例考虑结核球，余均提示结节性质待查。

**图 1:**
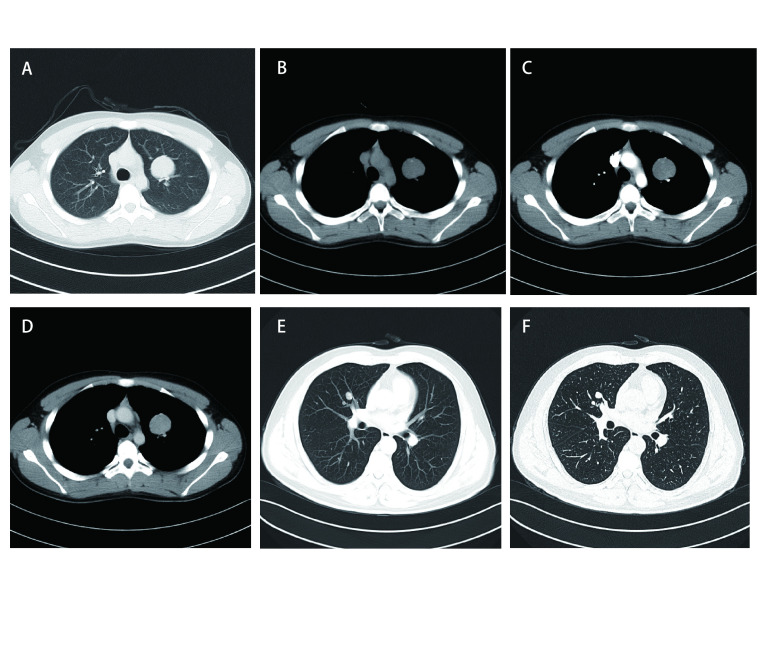
胸部CT：病灶典型。A、B、C、D为同一患者：左肺上叶见一类圆形肿块，边界清晰，内见点状钙化影，增强CT扫描均匀强化；E、F为同一患者（F为薄层CT）：右肺中叶见两个小结节，边界清楚，边缘见点状钙化。 Chest CT: lesions are typical. A, B, C and D are the same patient: A round mass is seen in the left upper lobe, with a clear boundary, punctate calcifications are seen inside, and the enhanced scan is evenly enhanced; E and F are the same patient (F is thin-slice CT): Two small nodules with clear boundaries and spotted calcifications on the edges are seen in the right middle lobe.

**图 2:**
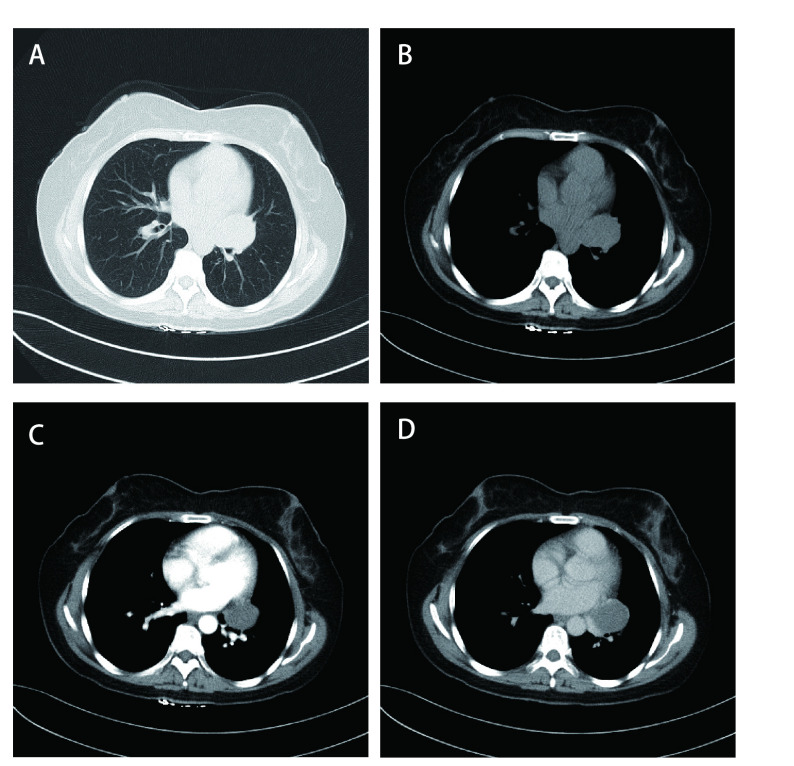
胸部CT：病变在肺门。A、B、C、D为同一患者：左肺门区见一密度增高团块影，边界较清。 Chest CT: lesions in the lung hilum. A, B, C and D are the same patient: A high density mass shadow was seen in the left hilar area, and the boundary was clear.

**图 3:**
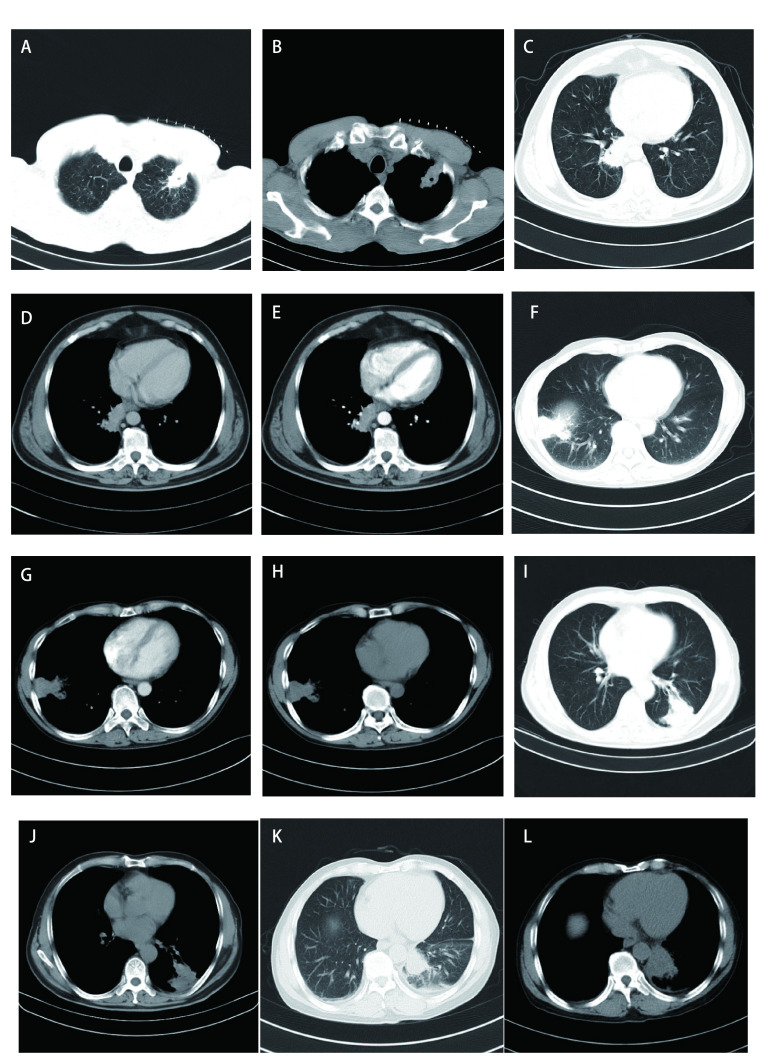
胸部CT：病灶边界不清楚。A、B为同一患者：左肺上叶见不规则片状密度增高影，内有小片低密度区；C、D、E为同一患者：右肺下叶后基地段脊柱旁见团块状密度增高影，呈分叶状，周围见短毛刺，增强扫描明显强化；F、G、H为同一患者：右肺下叶见一不规则片状高密度影，增强扫描呈不均匀轻中度强化；I、J为同一患者：左肺下叶背段及后基底段见一三角形软组织肿块，密度欠均匀，边界不清，内有多发点状、片状低密度影；K、L为同一患者：左肺下叶背段见一软组织肿块，最大截面约54 mm×49 mm，边界不清，密度欠均匀。 Chest CT: lesion boundary is unclear. A and B are the same patient: An irregular high density flake shadow with small low density area is showed in the left upper lobe; C, D and E are the same patient: CT showed a high density mass shadow in the posterior base segment of the right lower lobe, with unclear boundary, lobular shape, short burr around, enhanced CT scans significantly enhance; F, G and H are the same patient: An irregular high density flake shadow is showed in the right lower lobe, the enhanced scan is unevenly lightly moderately reinforced; I and J are the same patient: A triangular soft tissue lump shadow with multiple dots and flaky low density shadow was found in the dorsal and posterior basal segments of the left lower lobe, the density of lump shadow is uneven, the boundary is unclear; K and L are the same patient: A soft tissue lump shadow in the dorsal segment of left lower lobe, the maximum cross section size is about 54 mm×49 mm, the boundary is unclear and density is uneven.

**图 4:**
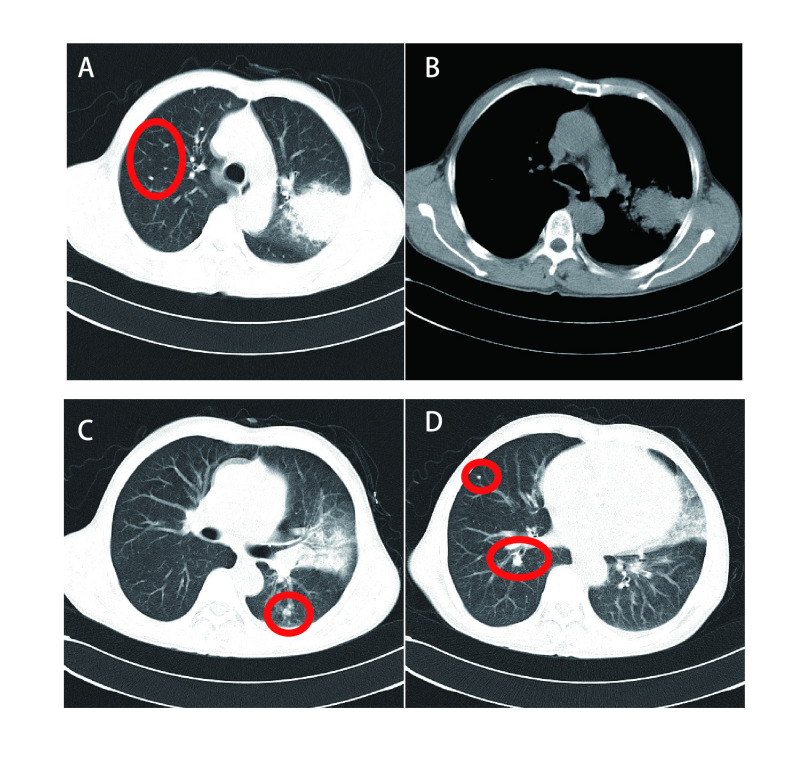
胸部CT：双肺多发病变、边缘不清。A、B、C、D为同一患者：左肺上叶后段、舌叶见大片密度增高影，边界模糊，密度不均，内有小片低密度影，余双肺见多发斑点状密度增高影（红色圈所圈部位），左侧少量胸腔积液。 Chest CT: multiple lesions and unclear edges. A, B, C, D are the same patient: A large high density flake shadow with small pieces of low density shadow is showed in the rear section and tongue of left lung upper, boundary blur, uneven density, the remaining double lungs see multiple spots high density shadow (red circle area), a small amount of chest fluid on the left.

### 1.4 术前诊断及手术方式

本组有12例术前行经皮肺穿刺组织病理活检术，有7例诊断为PSP（其中1例提示肺腺癌不能排除），5例提示良性病变。本研究共28例患者最终行手术治疗，24例行术中快速冷冻活组织病理切片检查，仅5例提示PSP，2例提示肺癌，1例良恶性不能判定，1例提示炎性病变，15例提示良性病变；25例行胸腔镜手术，3例行开胸手术；21例行肺部肿块楔形切除术，5例行肺叶切除术，1例行肺门肿瘤切除术，1例疑为肺癌故行纵隔、肺门及隆突下淋巴结清扫。7例未行手术治疗[其中1例行经皮肺穿刺确诊PSP并提示肺腺癌不能排除，骨发射计算机断层显像（emission CT, ECT）提示右髂骨转移瘤自行放弃手术治疗，出院后1个月于重庆某三甲医院行手术治疗，术后病理诊断腺癌合并PSP，髂骨诊断为外伤所致陈旧性病变；6例经皮肺穿刺确诊后签字出院]。

## 2 结果

### 2.1 病理检查结果

本组28例接受手术治疗的病例，最终术后病理结果1例诊断肺角化型鳞癌伴局部PSP，余均诊断为PSP。本组病理检查组织学特点（图 5）：镜下可见乳头状、实体性、硬化性及出血性4种不同的病理类型，至少包括有2种或2种以上病理类型。肿瘤细胞主要包括覆于乳头状结构表面的立方上皮细胞和位于实性区与乳头状结构之间的圆形间质细胞或者多角形细胞。14例行免疫组织化学检查均诊断为PSP（图 6），包括甲状腺转录因子1（thyroid transcription factor-1, TTF-1）、上皮膜抗原（epithelial membrane antigen, EMA）、波形蛋白（Vimentin）、天冬氨酸蛋白酶A（novel aspartie proteinase A, Napsin A）、增殖标志物ki-67（proliferation marker ki67, Ki67）、细胞角蛋白（cytokeratin, CK）、细胞角蛋白7（cytokeratin 7, CK7）、β-链蛋白（β-catenin），结果见表 2。

**图 5:**
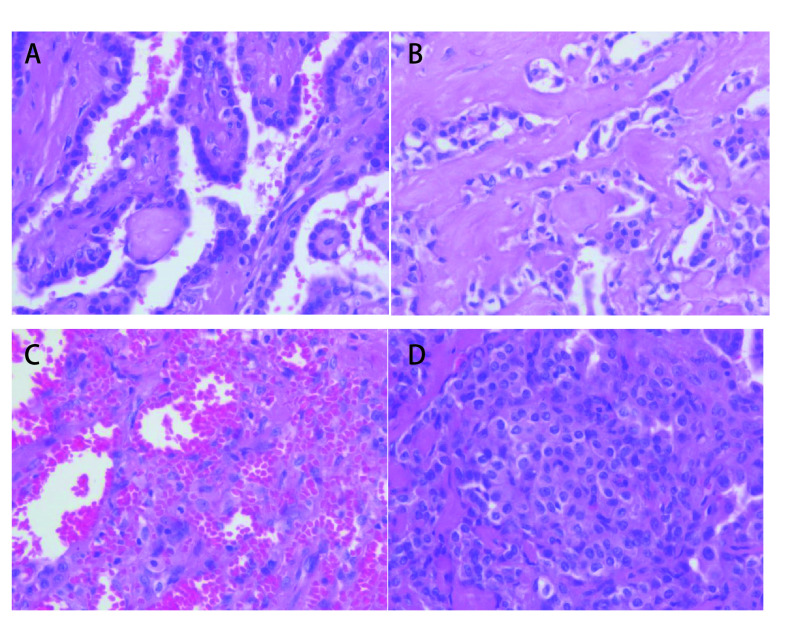
病理图片。乳头状结构（A）、硬化性结构（B）、出血区（C）、实性区（D）（HE染色，×400）。 Pathological picture. Papillary structure (A), sclerotic structure (B), hemorrhagic area (C) and solid structure (D) (HE staining, ×400).

**图 6:**
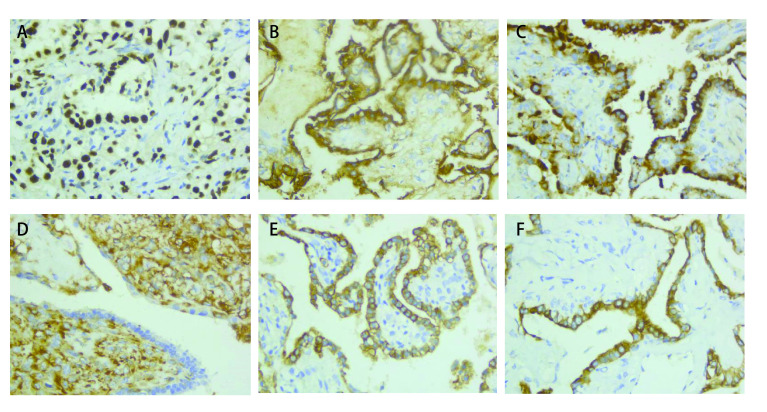
病理图片。A：TTF-1上皮和间质细胞（+）；B：EMA上皮和间质细胞（+）；C: Napsin A上皮细胞（+）；D：vimentin间质细胞（+）；E：CK-7上皮细胞（+）；F：CK上皮细胞（+）（免疫组化染色，×400）。 Pathological picture. A: TTF-1 epithelial cell and interstitial cell (+); B: EMA epithelial cell and interstitial cell (+); C: Napsin A epithelial cell (+); D: vimentin interstitial cell (+); E: CK-7 epithelial cell (+); F: CK epithelial cell (+) (immunohistochemistry staining, ×400).

**表 2:** 免疫组化结果 Immunohistochemical results

Immune marker	Epithelial cell positive	Interstitial cell positive
TTF-1	14	14
EMA	14	14
Vimentin	8	12
Napsin-A	8	0
β-catenin	5	6
CK	6	5
CK7	8	0
Ki67（1%+）	6	6
Ki67（2%+）	1	0
TTF-1: thyroid transcription factor-1; EMA: epithelial membrane antigen; Napsin-A: novel aspartie proteinase A; CK: cytokeratin; Ki67: proliferation marker ki67.		

### 2.2 预后及随访

本组行手术治疗患者预后：3例随访1年，4例随访2年，2例随访3年，6例随访5年，4例随访7年，3例随访8年，术后均恢复良好，均无复发和转移；1例随访5年，复查胸部CT无复发，最终因直肠癌死亡；1例左肺叶切除术后合并有乳腺癌，随访5年，复查胸部CT见右肺有2个小结节，患者拒绝再次手术治疗，后1年多未复查胸部CT，电话随访患者一般情况可，无呼吸道症状；4例失访。未行手术治疗患者预后：1例随访2年，2例随访3年，2例随访7年，复查未见肿块数目增多，肿块大小无明显变化，患者一般情况均良好；1例于我院诊断PSP，不能排除腺癌未予治疗，出院后1个月于重庆某三甲医院行手术治疗，术后病理诊断腺癌合并PSP，随访2年无复发及转移；1例失访。

## 3 讨论

### 3.1 临床特点

PSP是一种少见的肺部良性肿瘤，在1956年由Liebow和Hubbell首次提出^[1]^，过去被认为起源于内皮和血管组织，被称为“肺硬化性血管瘤（pulmonary sclerosing hemangioma, PSH）”。后经免疫组化结果证实PSP起源于未分化的呼吸道上皮组织（II型肺泡细胞），2015年世界卫生组织（World Health Organization, WHO）已将“硬化性血管瘤”这一术语重新更名为“硬化性肺泡细胞瘤”，归于一种亚型腺瘤^[2]^。PSP在4岁-70岁均可能发病，好发于亚洲非吸烟中年女性，男女发病比例为1:5^[3]^。本研究男女比例约1:2，可能与本组患者数量较少有关。PSP通常在行常规影像学检查中偶然发现，部分患者可出现咳嗽、咳痰、气促、咯血、胸痛、胸闷等非特异性症状，较为少见的临床表现为发热，如Zhou等^[4]^报道1例35岁女性患者间歇性发热1年余，胸部CT提示右肺多发圆形结节，边界清楚，密度均匀，行右肺中叶切除术治疗，术后病理诊断为PSP，手术治疗后患者无再发热症状。

PSP是良性肿瘤，有报道其可转移至其他部位，如胸膜播散^[5]^、胃转移^[6]^、骨转移^[7]^、淋巴结转移^[7-9]^，其中以淋巴结转移多见，转移率约为2%-4%^[10]^；转移至胃、胸膜、骨较为少见。Suzuki等^[5]^报告1例PSP合并胸膜播散的病例：1例57岁女性患者行胸部CT检查示右肺下叶见一直径2.5 cm的肿块，经支气管镜活检未能明确诊断，后行右肺下叶及胸膜小结节切除术，最终术后病理证实为PSH伴胸膜播散。Bae等^[6]^报道1例PSP转移至胃患者，自首次发现病灶开始，随访3年肺内病灶生长缓慢，胃内转移结节大小未见明显变化。Kim等^[7]^报道了1例73岁女性行胸部CT提示右肺下叶孤立圆形肿块影，伴有淋巴结增大，经皮肺穿刺病理活检后，诊断为PSH，3年后再次行CT扫描显示肺部肿块增大并支气管周围及纵隔多发淋巴结病变，行右中下肺叶切除术及淋巴结清扫术后病理检查诊断为PSP，其中18个淋巴结中有3个淋巴结转移，转移的淋巴结的组织学和免疫组织化学特征与肺部肿瘤相同，6个月后，患者行脊柱磁共振成像（magnetic resonance imaging, MRI）发现腰椎病变，行细针穿刺进行组织学、免疫组织化学染色检查与PSP典型特征相符，患者接受了L2-S2脊柱的放疗，自2003年发病开始随访至2015年无复发。由此可见，PSP即使可转移至其他远处器官，但肿瘤生长较为缓慢，复发也是非常罕见的，故PSP被认为是肺部良性肿瘤。PSP转移的机制目前仍不清楚，有学者^[11, 12]^提出PSP淋巴结转移可能与年龄、性别、原发肿瘤位置和肿瘤大小有关，认为转移更可能发生于年轻、男性、肺下叶、直径较大肿瘤的患者。也有学者^[13]^认为Vimentin在上皮细胞的迁移和侵袭中起着重要的作用，PSP转移可能与Vimentin表达的高水平有关。Sun等^[14]^认为圆形细胞可能源自表面细胞的上皮-间充质转化（epithelial-mesenchymal transition, EMT），EMT可能与恶性肿瘤转移密切相关^[15]^，Wang等^[9]^发现转移性病灶主要由圆形细胞组成，因此，PSP转移机制可能与EMT相关。Cho等^[16]^认为基质金属蛋白酶9（matrix metalloproteinase-9, MMP-9）在肺PSP中表达的升高可能与转移或播散有关。

PSP同时合并其他类型的肺部肿瘤较为少见。有病例报道腺癌和PSH存在于同一个肺结节内，结节分为两个独立的部分，分别表现出典型的PSP和腺癌的组织学特征^[17]^。LuLu^[18]^ 报道了1例74岁的女性患者在同一肺的右叶同时发生2种罕见肿瘤，分别是孤立的右肺上叶肺肉瘤样癌（pulmonary sarcomatoid carcinoma, PSC）和右肺下叶PSP。Wang等^[19]^报道了1例有类癌的肺叶中同时发生的PSP病例。支气管乳头状瘤和PSP也可以同时发生^[20]^。本研究中亦有2例PSP女性患者最终术后病理诊断分别合并肺角化型鳞癌和腺癌。目前PSP与其他肺部肿瘤共存的机制尚不清楚。PSP可能与肺腺癌存在相同的分子基因改变，说明两者间或许有一定的关联^[21]^，不排除是二者共同存在的基因基础。总之，临床医师在诊治该病时，需要警惕PSP同时合并其他肺部肿瘤可能。

### 3.2 病理特点

PSP包括两种基本类型的肿瘤细胞：表面立方上皮细胞和圆形间质细胞（多角形细胞）^[3, 22]^。病理上主要包括4个区域（乳头区、实性区、硬化区、出血区），PSP至少由2种或者2种以上区域成分组成，大多数由3种以上成分组成。免疫组织化学结果表明TTF-1和EMA均为阳性^[3]^。本组14例行免疫组化检查，TTF-1、EMA在上皮和间质细胞中均阳性，CK7和Napsin-A在表面立方体细胞中为阳性，而在圆形基质细胞中为阴性，与文献报道相符合。Yang等^[23]^回顾性分析了59例PSP病例，术中冷冻切片的诊断准确率为44.1%，通过分析术中冰冻活组织切片误诊病例，总结了术中冰冻切片易将PSP误诊的5个病理陷阱，包括肿瘤细胞过度增生、腺样结构、纤维组织增生样硬化、凝固性坏死和细胞异型性。总之，诊断PSP的关键是识别肿瘤中双细胞群体，即表面立方细胞和圆形间质细胞。PSP的分子病理改变尚不十分清楚。杂合性丢失（loss of heterozygosity, LOH）分析的研究^[24]^表明PSP与细支气管肺泡癌（bronchioloalveolar carcinoma, BAC）之间的等位基因缺失模式相似：5q和10q处的LOH以及p16位点处的LOH；这种相似性进一步支持了这两组肿瘤的共同起源的假说，但并不能证明PSP是恶性肿瘤。蛋白激酶B（protein kinase B, Akt1）是一种丝氨酸/苏氨酸激酶，可刺激多种癌症相关过程，包括细胞增殖、存活和生长，Nasr等^[25]^报告1例PSP儿童有磷酸酶和张力蛋白同源物（phosphatase and tensin homolog deleted on chromosome,  *PTEN*）基因突变，该*PTEN*基因通过对抗磷脂酰肌醇3-激酶/蛋白激酶B/雷帕霉素靶蛋白（phosphatidylinositol-3-kinase/protein kinase B/mammalian target of rapamyoin, PI3K/AKT/mTOR）通路调节细胞生长。Jung等^[26]^对44例PSP进行了深入的全外显子测序，发现45.6%的病例在*AKT1*中具有复发性体细胞突变（或拷贝数改变），其中大多数是*AKT1* p.E17K突变，此突变可能是形成PSP的最常见的驱动程序改变，另有4.5%的病例显示*β-catenin*突变。与肺腺癌相比，PSP基因组仅包含一个驱动基因突变（*AKT1*或*β-catenin*），这可能为了解PSH的良性生物学和肺恶性肿瘤的差异基因组诊断提供线索。Fan等^[27]^通过全外显子组测序鉴定了1例多发性病变的PSP女性患者的15个体细胞突变基因：多种表皮生长因子样结构域蛋白6（multiple epidermal growth factor-like domains protein 6, *MEGF6*）、动力蛋白轴索重链（dynein axonemal heavy chain, *DNAH*）、*AKT1*、G蛋白调节的神经突生长诱导物2（G-protein-regulated inducer of neurite growth 2, *GPRIN2*）、磷酸肌醇-3-激酶衔接蛋白1（phosphoinositol-3-kinase adaptor protein 1, *PIK3AP1*）、F-box 40蛋白（F-box 40 protein, *FBXO40*）、泛素连接酶蛋白1（ubiquitin ligase protein 1, *HERC1*）、液泡蛋白分选复合体亚基16（vacuole protein sorting 16,  *VPS16*)、膜占据和识别关系蛋白1 （membrane occupation and recognition nexus protein 1, *MORN1*）、锌指474（zinc finger 474, *ZNF474*）、连环蛋白β1（catenin β1, *CTNNB1*）、*ZNF251*、结节性硬化复合物1（tuberous sclerosis complex 1, *TSC1*）、共济失调毛细血管扩张突变体（ataxia-telangiectasia mutant, *ATM*）、激酶插入域包含受体（kinase-insert domain-containing receptor, *KDR*）基因；通路分析表明PI3K/AKT信号通路可能存在*CTNNB1*、*AKT1*、*TSC1*的突变，在血管内皮生长因子（vascular endothelial-derived growth factor, VEGF）信号途径中可能存在*AKT1*、*KDR*、*ATM*的突变，因此，AKT1的激活可能在PSP中发挥着重要作用。Jiang等^[28]^通过二代测序（next-generation sequencing, NGS）和桑格测序鉴定出在PSP肿瘤组织中的两个突变：*AKT1* E17K和原癌基因*BRAF* V600E，这是PSP患者中*BRAF* V600E突变的第1例病例报告，这一发现可增加我们对PSP发病机制的理解，并暗示了在这种少见肿瘤类型中需要对*BRAF* V600E进行进一步测试的必要性。Lee等^[21]^通过分析了肺部PSP和腺癌的临床病理特征（微卫星改变和CpG岛甲基化），以比较其分子异常模式，结果表明PSP和腺癌具有相似的遗传和表观遗传畸变，但在抑癌基因中表现出显著差异。*p53*是一种抑癌基因，其突变以及p53相关的蛋白积累与恶性肿瘤的发生和临床进展相关。在15.8%（3/19）的病例中观察到p53蛋白的表达和基因突变，在多角形细胞中的表达水平和突变率高于表面长方体细胞，这可能有助于解释PSH偶尔浸润和转移的临床发现^[29]^。

### 3.3 影像学特点

PSP典型病例在X线平片或CT平扫上常常表现为孤立性、边界清楚的圆形或类圆形结节或肿块，密度均匀，增强扫描后多为均匀性强化，肿块无肺叶分布优势^[30]^。朱靓等^[31]^报道15例PSP均为单发小结节，病灶均小于30 mm。本组单发病灶30例（比例约为86%），病变位于右肺与左肺的比例为3:4，肿块平均直径34 mm，与文献报道基本相符合。PSP除了上述典型的影像学特征，还有血管贴边征、尾征、空气新月征、晕征、抱球征等影像学特征^[31-33]^，多层螺旋计算机断层扫描（multi slice spiral computed tomography, MSCT）经多方位重组（multi planner reformation, MPR）、容积演示（volume rendering, VR）三维重建技术处理后可清晰显现这些特征的征象^[33]^。但值得注意的是类似表现亦可在周围型肺癌、错构瘤、炎性假瘤、肺结核球、肺曲霉球、转移瘤、原发性肺腺癌、类癌中出现。氟脱氧葡萄糖（fluorodeoxyglucose, FDG）是正电子发射计算机断层扫描（positron emission CT, PET-CT）最常用的示踪剂之一，其能量摄取程度与肿瘤的恶性程度相关。PSP通常表现为FDG摄取低至中等，这与其生长缓慢相对应。Xu等^[34]^研究表明在典型的PSP病例中，FDG摄取量与肿瘤大小呈正相关，FDG摄取量与非典型PSP大小之间没有相关性，有症状的患者显示FDG的平均摄取量高于无症状组。PSP伴随炎症、结核等FDG摄取量会增加。

### 3.4 诊断及鉴别诊断

PSP的误诊、误治在临床上较为常见。PSP可通过经皮肺穿刺术、经支气管镜内超声引导细针针吸术、术中快速冰冻组织切片等病理检查明确诊断。本组24例术中行快速冷冻活组织病理切片，仅5例提示PSP；12例行经皮肺穿刺，7例诊断为PSP；可见术前病理诊断困难，误诊率高。因此，绝大多数PSP需行术后病理组织检查才能明确诊断。

PSP需要与下列疾病进行鉴别诊断：①周围型肺癌：病灶进展迅速，增强扫描时病灶呈明显不均匀强化，常常可见毛刺、分叶、胸膜凹陷征及细支气管充气征，增强扫描时强化程度较PSP低，且强化多不均匀；分叶征、动静脉期强化率有助于鉴别；②错构瘤：多发于男性，出现典型的“爆米花”样钙化与脂肪成分有助于诊断错构瘤，增强扫描时强化多不明显，而PSP钙化多呈砂砾样、点状，增强扫描呈明显强化，难以鉴别者需要病理证实；③肺结核球：好发于肺尖与下叶背段，可有发热、盗汗等中毒症状，CT多表现为类圆形结节影，密度不均匀，可出现坏死病灶，常伴卫星灶，增强扫描结核球强化程度较PSP弱；④肺曲霉球：病灶多在空腔或空洞最低处，可随体位变动而移动，增强扫描多无强化；⑤炎性假瘤：多有肺炎病史，影像学上病灶边界不清楚，密度不均匀，形态不一，不均匀强化，多抗感染治疗有效；⑥转移瘤：多有原发肿瘤病灶，影像学一般为多发结节，PET-CT有助于鉴别。

### 3.5 治疗及预后

本组28例行手术治疗，术后恢复良好，多数患者术后1年-8年无复发及转移，可见手术仍是目前治疗PSP首选的治疗方法；本组最终有6例未行手术及其他任何治疗，随访2年-7年，5例一般情况良好，复查病变无明显增大、增多，因此，部分PSP患者或许可长期带瘤生存，仅需定期复查。PSP发病可能与性激素受体有关，多数PSP患者雌激素受体（estrogen receptor, ER）与孕激素受体（progesterone receptor, PR）均为阳性^[35]^，但PSP是否可行内分泌治疗尚需进一步探究。

总之，本研究通过对35例PSP患者的胸部CT、病理特点等临床特征进行回顾性分析，更深一步认识PSP，该病即使发生转移，但生长缓慢，总体预后良好，故仍被认为是一种肺部良性肿瘤，临床上较为少见，好发于中年女性，影像学及临床表现缺乏特异性，术前病理检查易误诊，多数患者需术后病理才能确诊。
